# Green synthesis of metal nanoparticles using microorganisms and their application in the agrifood sector

**DOI:** 10.1186/s12951-021-00834-3

**Published:** 2021-03-26

**Authors:** Howra Bahrulolum, Saghi Nooraei, Nahid Javanshir, Hossein Tarrahimofrad, Vasighe Sadat Mirbagheri, Andrew J. Easton, Gholamreza Ahmadian

**Affiliations:** 1 Department of Industrial Environmental and Biotechnology, National Institute of Genetic Engineering and Biotechnology (NIGEB), P.O.BOX: 14155-6343, 1497716316 Tehran, Iran; 2 Department of Animal Biotechnology, National Institute of Genetic Engineering and Biotechnology (NIGEB), Tehran, Iran; 3 Faculty of Fisheries and Environment Science, Gorgan University of Agriculture Science and Natural Resources, Gorgan, Iran; 4 School of Life Sciences, Gibbet Hill Campus, University of Warwick, Coventry, UK

**Keywords:** Agriculture, Metal nanoparticles, Green synthesis, Microorganisms, Nanopesticides, Nanofungicides, Nanofertilizers, Nanobiosensors, Crop protection

## Abstract

The agricultural sector is currently facing many global challenges, such as climate change, and environmental problems such as the release of pesticides and fertilizers, which will be exacerbated in the face of population growth and food shortages. Therefore, the need to change traditional farming methods and replace them with new technologies is essential, and the application of nanotechnology, especially green technology offers considerable promise in alleviating these problems. Nanotechnology has led to changes and advances in many technologies and has the potential to transform various fields of the agricultural sector, including biosensors, pesticides, fertilizers, food packaging and other areas of the agricultural industry. Due to their unique properties, nanomaterials are considered as suitable carriers for stabilizing fertilizers and pesticides, as well as facilitating controlled nutrient transfer and increasing crop protection. The production of nanoparticles by physical and chemical methods requires the use of hazardous materials, advanced equipment, and has a negative impact on the environment. Thus, over the last decade, research activities in the context of nanotechnology have shifted towards environmentally friendly and economically viable ‘green’ synthesis to support the increasing use of nanoparticles in various industries. Green synthesis, as part of bio-inspired protocols, provides reliable and sustainable methods for the biosynthesis of nanoparticles by a wide range of microorganisms rather than current synthetic processes. Therefore, this field is developing rapidly and new methods in this field are constantly being invented to improve the properties of nanoparticles. In this review, we consider the latest advances and innovations in the production of metal nanoparticles using green synthesis by different groups of microorganisms and the application of these nanoparticles in various agricultural sectors to achieve food security, improve crop production and reduce the use of pesticides. In addition, the mechanism of synthesis of metal nanoparticles by different microorganisms and their advantages and disadvantages compared to other common methods are presented.

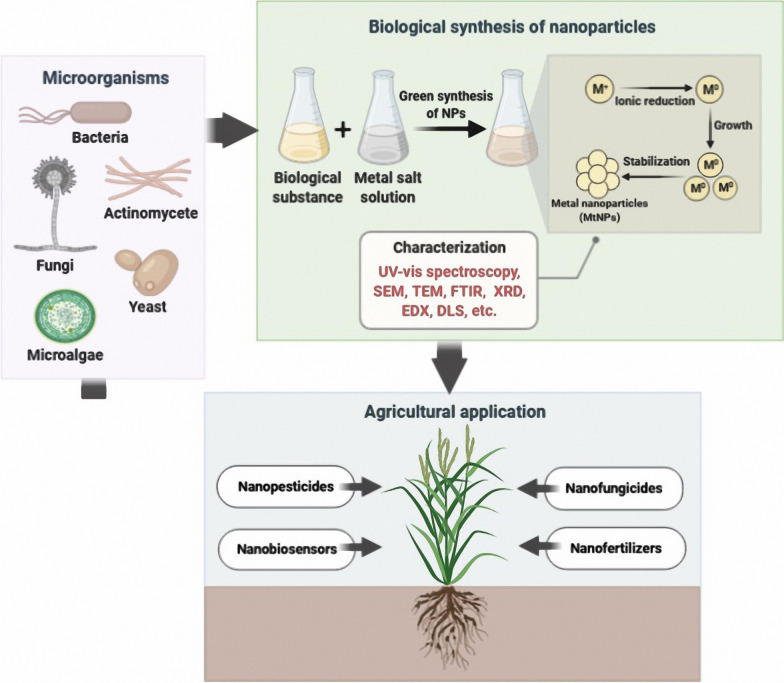

## Background

Nanoparticles now play a key role in most technologies, including medicine, cosmetics, agriculture and the food sciences [1]. Recently, the synthesis of metal nanoparticles (MtNPs) using microorganisms and plants has been recognized as an efficient and green method for further exploitation of microorganisms as nanofactories [2]. Given the challenges facing the international community, especially in terms of population growth and climate change, nanotechnology can have positive effects on improving the quality of agricultural products, minimizing the adverse effects of agricultural pesticides on the environment and human health, and increasing productivity and food security. Unique properties of nanoscale materials make them an excellent candidate for using in the design and development of new tools for supporting agriculture and related industries. Nanotechnology can improve agricultural processes such as soil quality and the quality of agricultural products by using nanoparticle-based fertilizers or by stimulating plant growth. In addition, the use of fertilizers and pesticides using nanoparticle-based carriers and compounds is reduced without reducing productivity [3]. Nanotechnology can also minimize waste by fabricating products that are more efficient. Applications of nanosensor technology can lead to the development of precision agriculture and efficient management of resources, including energy and materials used [4]. In particular, the goal of developing green nanotechnology, which utilizes biological pathways for the synthesis of nanomaterials is minimizing the production of hazardous substances. Meanwhile, the amount of energy input in green nanotechnology is much lower than in other technologies; almost no toxic chemicals are produced during synthesis, and their environmental compatibility is very high. Therefore, green nanomaterials produced can be widely used in various industries [5]. Depending on the application required, different types of nanomaterials are used in agriculture. For example, for use in pesticides, nanoparticles are used as carriers, which gradually release the active ingredient(s) to reduce their overall consumption. When the goal is to improve the packaging of agricultural products, the nanomaterials used are selected to be biocompatible and do not have negative effects on human health while increasing the shelf life of food. Alternatively, high-sensitivity nanosensors with plasmonic properties such as silver or gold nanoparticles can be used to measure environmental conditions, report changes in a timely way, and intelligently control plant needs in greenhouses. In all cases, the small size and unique physical and chemical properties of the MtNPs make them attractive for use in various agricultural sector [1]. To date, a broad range of nanotechnology applications have emerged in the agrifood sector, such as nanosensors, tracking devices, targeted delivery of required components, food safety and intelligent packaging which can affect different aspects of our lives [6–8].

Several advanced techniques are available to improve precision breeding methods and enable precise control of the green synthesis process at the nanometer scale. Nanotechnology can also be an alternative source for generating fertilizer, as MtNPs have been shown to be able to increase germination in agricultural seeds. Other applications include the use of nanoscale carriers for effective delivery of fertilizers, pesticides, plant growth regulators, and other similar compounds. These processes improve the stability of these materials to environmental degradation and ultimately reduce their amount used, which in turn leads to reductions in chemical runoff and associated environmental problems. Carriers can also be designed to increase the communication between plant roots and the surrounding soil structure [9]. Modified nanoparticles can be added to conventional fertilizers for improving nitrogen storage capacity which leads to reduced nitrogen loss and better nutrition for agricultural products. Several nanoemulsions have also been formulated to increase the biological compatibility of herbicides and pesticides [10].

Microorganisms are important nanofactories that are able to accumulate and detoxify heavy metals due to the presence of various reductase enzymes that are able to reduce metal salts to MtNP [2]. In recent research, bacteria such as *Pseudomonas* deptenis [11], *Visella* *oriza* [12] *Bacillus* methylotrophicus [13], *Bhargavaea* *indica* and *Brevibacterium* *frigoritolerans* have been shown to be able to synthesize silver (Ag) and gold (Au) nanoparticles. MtNPs have also been synthesized by various genera of microorganisms such as *Lactobacillus,* *Bacillus*, *Pseudomonas*, *Streptomyces,* *Klebsiella*, *Enterobacter*, *Escherichia*, *Aeromonas*, *Corynebacterium*, *Weissella*, *Rhodobacter*, *Rhodococcus*, *Brevibacterium*, *Trichoderma*, *Desulfovibrio*, *Sargassum*, *Shewanella*, *Plectonemaboryanum*, *Pyrobacul*um and *Rhodopseudomonas* [2]. The synthesis of nanoparticles by actinomycetes has not yet been well studied, although studies to date have shown that nanoparticles produced by actinomycetes have very good dispersion and stability and have significant lethal activity against various pathogens [14]. In particular, various microorganisms, such as bacteria, fungi, yeasts and microalgae have been shown to produce MtNPs either intra- or extracellularly. These microorganisms are able to produce organic matter inside, and to transport it to the outside of their cells [15]. Microorganisms as nanofactories have great potential as environmentally friendly, inexpensive, and non-toxic tools that do not require much energy for MtNPs synthesis compared to physicochemical methods. Among the various mechanisms for the green synthesis of MtNPs, those that perform extracellular synthesis are of great interest because the extracellular location of the material eliminates the need for costly and complex downstream processing steps to recover intracellular nanoparticles [2]. Green synthesis of MtNPs using microorganisms has several advantages compared to conventional physicochemical methods. In particular it offers a rapid, cost-effective, clean, non-toxic and environmentally friendly method for the synthesis of MtNPs with a wide range of sizes, shapes, compositions and physicochemical properties [16, 17]. However, the main drawbacks of microorganism-based synthesis of MtNPs includes complicated steps such as microbial sampling, isolation, culturing and storage. In addition, the recovery of MtNPs produced by this method requires downstream processing [2].

In this review, we explore the various potential applications of green synthesized MtNPs with an emphasis on agriculture. This includes consideration of advantages of green synthesis of MtNPs using different microorganisms.

## Green synthesis of MtNPs by microorganisms and their characterization

Various approaches have been used for MtNP synthesis, such as physical, chemical, and biological methods. The physical and chemical methods for MtNP synthesis have many disadvantages including the use of expensive equipment, high heat generation, high energy consumption and low production yield [18, 19]. The main drawback of these methods is the use of toxic chemicals, which present several environmental problems [19, 20]. This has generated a need for an environmentally friendly option for the synthesis of MtNPs, the current focus of which is the green synthesis of MtNPs from biological routes such as microorganisms, plants, microbial enzymes, polysaccharides and degradable polymers [21]. Green synthesis methods are more beneficial than traditional physical and chemical methods because they are simple, cost-effective, free of toxic and environmentally unfriendly chemicals, and as a result they have gained considerable importance in recent years [20].

The innovative and diverse applications of MtNPs in various fields including medical sciences, environmental sciences and agriculture, research on MtNPs and different approaches of their synthesis has increased rapidly over recent years [18, 22]. The synthesis of MtNPs is generally performed using one of two different approaches, broadly considered as top-down and bottom-up approaches. In top-down approaches, bulk materials are broken down into nano-sized particles to form MtNPs, based on their reduction in size, using various physical and chemical techniques [18, 23]. The main drawback of this method is the production of nanoparticles with imperfect surface structures. Also, it is an expensive and time consuming approach so it is not appropriate for large-scale production [23]. In bottom-up approaches, nanoparticles are produced by self-assembly of structures at the atomic and molecular scales, resulting in a more precise size, shape and molecular composition [24]. This method includes chemical and biological methods of production [18].

**Fig. 1:**
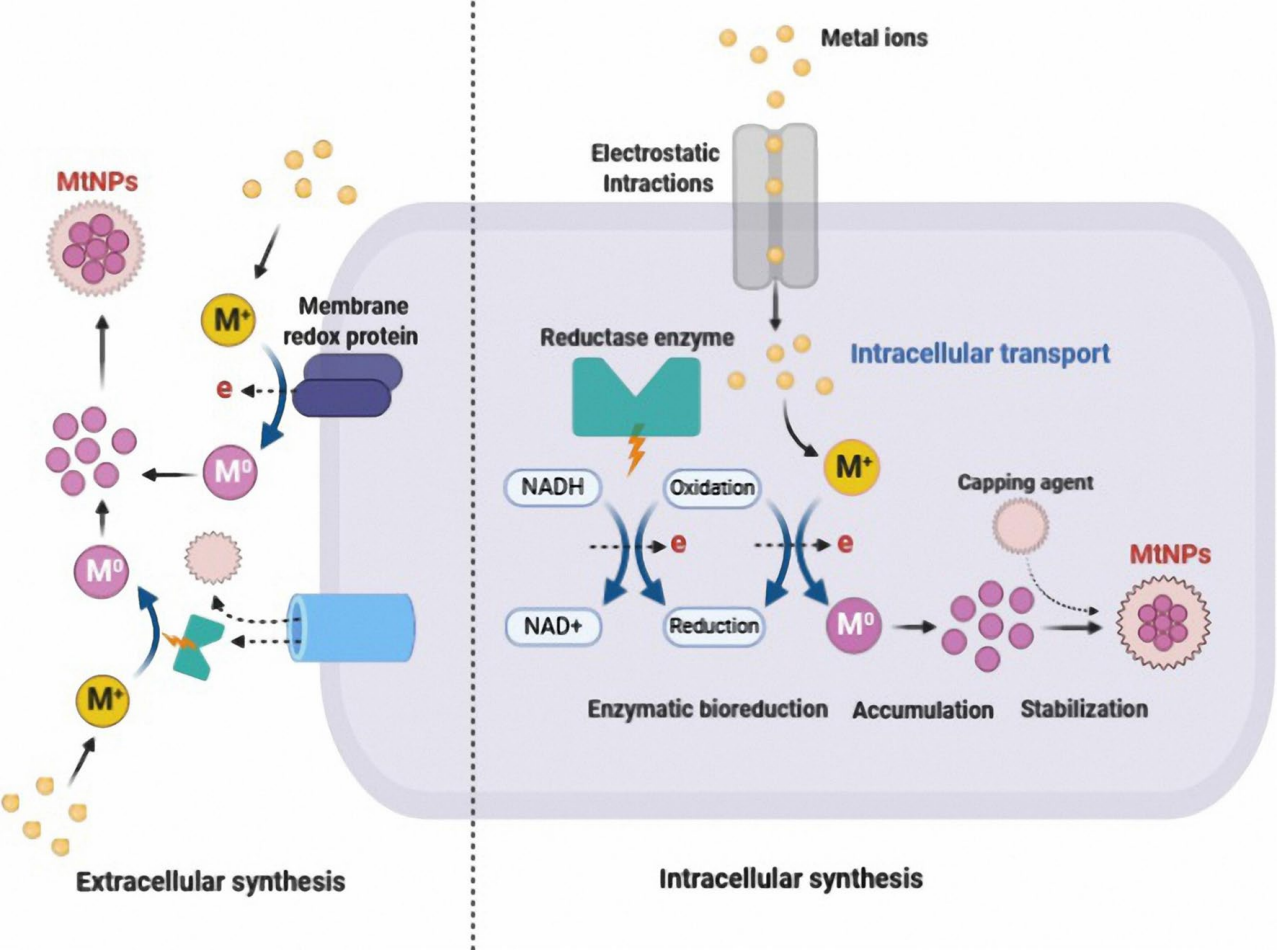
Schematic representation of the mechanisms of extracellular and intracellular biosynthesis of MtNPs. Extracellular biosynthesis of MtNPs carried out by trapping metal ions on the cell wall and reducing them in the presence of secreted enzymes or metabolite. In the intracellular biosynthesis of MtNPs, after transfer of metal ions into cell cytoplasm, the metal ions are reduced as a result of metabolic reactions with enzymes, such as nitrate reductase

Among the various biological sources for the green synthesis of MtNPs, green synthesis mediated by microorganisms has acquired a special place due to their high growth rate, ease of cultivation and ability to grow in ambient conditions of temperature, pH and pressure [25]. Different microorganisms can serve as potential biofactories for the eco-friendly and inexpensive synthesis of various MtNPs containing metals such as silver, gold, copper, zinc, titanium, palladium and nickel. This can be achieved to generate MtNPs with a defined shape, size, composition and monodispersity of particles [18, 22, 26]. The biosynthetic mechanism of MtNPs in microorganisms can be carried out by trapping target metal ions from the surrounding environment and enzymatically converting them into elemental form, following a reduction mechanism [26]. Not all microorganisms are able to produce MtNPs because they are produced through metabolic pathways and through cellular enzymes that may not be present in some organisms. The synthesis of MtNPs also is dependent on the capacity of microorganisms for tolerating heavy metals. High metal stresses can affect various microbial activities and some microorganisms are able to reduce metal ions to the respective metals under stress condition. In general, microorganisms that live in metal-rich habitats are highly resistant to those metals due to their uptake and chelation of by intracellular and extracellular proteins. Consequently, this method, which mimics the natural bio-mineralization process, could be a favorable approach for the MtNPs synthesis [27]. Figure 1 shows a schematic illustration of intracellular and extracellular mechanisms of MtNPs biosynthesis. Intracellular biosynthesis involves unique transport systems in microorganisms in which the cell wall plays an important role due to its negative charge: positively charged metal ions are deposited in negatively charged cell walls through electrostatic interactions. After transport into the cells of the microorganism, ions are reduced using metabolic reactions mediated by enzymes such as nitrate reductase to forms MtNPs. The MtNPs accumulated in the periplasmic space can then be passed through the cell wall [28, 29].

The extracellular biosynthesis of MtNPs is also a nitrate reductase-mediated synthesis in which the MtNPs are produced by reductase enzymes which are either located in the cell wall or secreted from the cell to the growth medium. In this process the nitrate reductase reduces metal ions to the metallic forms [27, 29].

The presence of diverse components such as enzymes, proteins, and other biological molecules in microorganisms also play an important role in the process of reducing MtNPs [27]. Studies have shown that NADH-dependent enzymes are responsible for the MtNP synthesis. The reduction mechanisms seem to begin by transferring an electron from NADH by NADH-dependent reductases as the electron carrier [30]. In addition, proteins secreted by microorganisms can act primarily as a stabilizing agent and provides colloidal stability while preventing agglomeration of MtNPs [27].

For intracellular synthetic approaches microorganisms are cultured in a suitable growth medium with favorable pH and temperature conditions [23]. The biomass is harvested after an optimal incubation period and washed thoroughly with sterile water to minimize potentially undesirable effects of the culture medium. The resulting biomass is then incubated with metal salt solution. In addition to the use of whole microorganisms for intracellular synthesis of MtNPs an alternative is the use of cell-free (CF) approaches using either culture supernatant or cell-free extracts (CFE) [22]. In the CF approach using medium supernatant, after culturing the microorganisms in a liquid culture medium, the mixture containing the culture medium and biomass is centrifuged and the supernatant collected and incubated with an aqueous metal salt solution to synthesize the MtNPs. In this method, the compounds of the culture medium containing the appropriate enzymes and other essential secretory components produced by the microorganism are used to synthesize the MtNPs and also to act as reducing and capping agents. In approaches using cell-free extracts, the microorganisms are removed from the culture medium and resuspended in sterile distilled water for an approriate time. The resulting CFE is collected after centrifugation and is incubated with metal salt solutions, leading to the generation of MtNPs. In this approach the microorganisms and culture medium are removed through repeated washings, and only biomolecules released by cells due to autolysis or starvation conditions mediate synthesis of the MtNPs [19, 22, 25, 31]. In all cell free processes a color change in the reaction mixture is frequently the first indication of nanoparticle synthesis with the color change being dependent on the precise nature of the MtNP being produced. For example, a change in color from pale yellow to dark purple indicates the formation of gold nanoparticles, a pale yellow to deep brown color change indicates the formation of silver nanoparticles and a yellow to yellowish-white color change indicates the formation of manganese and zinc nanoparticles [19, 25, 32].

Various physiological factors including microbial source, reaction temperature, pH, pressure, incubation time and metal salt concentration affect the synthesis of various MtNPs. Optimization of these physiological parameters is required for synthesis of nanoparticles with accurate size, morphology and chemical compositions [33, 34]. After synthesis of MtNPs, purification before their use in any application is essential. Typically, repeated washing and high-speed centrifugation are performed to separate and enrich the produced MtNPs and to eliminate unreacted bioactive molecules [34]. In-cell synthesized nanoparticles require additional purification steps such as ultrasonication or reaction with appropriate detergents, which release the MtNPs after breakdown of the cell wall. These additional steps reduce the economic benefit of this approach [19].

Characterization of MtNPs synthesized from microorganisms is performed using various analytical techniques. UV–visible spectroscopy is generally used to confirm the synthesis and stability of MtNPs. Fourier-transform infrared (FTIR) spectroscopy is used to measure the properties of MtNPs such as chemical concentration, surface chemistry, surface functional groups and atomic arrangement [33] and transmission electron microscopy (TEM), scanning electron microscopy (SEM) and atomic force microscopy (AFM) can be used to visualize the position, size and morphology of MtNPs [35]. X-ray powder diffraction (XRD) is used to determine the crystallographic structure [33]. The elemental composition of MtNPs is usually examine by energy dispersive x-ray spectroscopy (EDS) [36]. Dynamic light scattering (DLS) method is mainly used to evaluate the size as well as surface charge of MtNPs [33].

## Application of green synthesized MtNPs in agriculture

Green-synthesized MtNPs have many potential applications in agriculture to increase the productivity of agricultural products. MtNPs are commonly used for generating products such as nanopesticides, nanofungicides, nanobiosensors and nanofertilizers. These nano-based products can help increase the quality and yield of agricultural products, reduce chemical pollution or even protect crops from environmental pressures [37].

The use of biosensors has revolutionized agricultural systems to increase the production of quality agricultural products due to their ability to quickly identify pathogens as well as their powerful monitoring and analytical capabilities [38]. Nanobiosensors are a modified version of a biosensor that can be described as an analytical unit by incorporating a biological sensitive element with a physicochemical transducer [39]. Nanobiosensors including enzymatic biosensors, genosensors, aptasensors, and immunosensors are made using a wide range of electrochemical, biological or physicochemical transducers. The use of these sensors has received much attention due to their fast, specific and selective performance in detection of toxins and plant pathogens [38]. Pesticides are used to protect plants from harmful agents such as plant pathogens and insects, to increase crop yield [40]. One of the most important challenges of using existing chemical pesticides is their negative effects on agricultural products in the food chain and ultimately on human health [37].

Nanopesticides represent an emerging nanobiotechnological development to encapsulate pesticides for controlled release and to improve the selectivity and stability of pesticides [37, 41]. These nanopesticides can offer a wide range of benefits including increased efficiency, durability and reduced amount of active ingredient required in their formulation [42, 43]. The nano-formulation of pesticides with MtNPs has shown a stronger effect against phytopathogens, insects and other pests that threaten crops. Fungi are the most common plant pathogens and cause more than 70% of major crop damage [40, 44]. To control this damage common fungicides are currently used, the widespread use of which for long-term disease management leads to environmental pollution and dangerous effects on the ecosystem. The use of nanofungicides is an effective strategy against fungal pathogens. The use of MtNPs in the formulation of nanofungicides is the most common of their applications. These nanofungicides offer targeted delivery and greater bioavailability due to higher solubility and permeability, lower doses, lower dose-dependent toxicity, and controlled release [45].

Fertilizers are natural or synthetic substances that contain chemical elements necessary to improve plant growth and productivity and improve natural fertility by overcoming micronutrient deficiencies. The main problem of excessive and long-term use of chemical fertilizers in the agricultural sector is the reduction of soil fertility, which ultimately affects the production of agricultural products. Nanofertilizers are environmentally friendly fertilizers or smart fertilizers that deliver nutrients in small but effective amounts to plants. Nutrient uptake can be increased by encapsulating nanofertilizers, which ultimately reduces nutrient loss, promotes proper plant growth and improves crop quality [40, 41, 44]. Nano-formulations provide gradual and controlled release of nutrients to the target sites through direct internalization of products, which prevents nutrients from interacting with soil, water, air and microorganisms resulting in minimizing the risk of environmental degradation [43]. It has been frequently observed that the use of MtNP-based nanofertilizers has significant potential to increase crop productivity.

The application of synthesized green nanoparticle technology in the food or agricultural sector gives flexibility to conventional crop production systems, as it allows the controlled release of pesticides and fertilizers, as well as the targeted delivery of biological molecules. Interactions between MtNPs and plant responses are manifested by increase in breeding, and ultimately, it improves the quality and productivity of products [46]. In the following subsections, different species of microorganisms used for biosynthesis of MtNPs, and their perspective in agricultural applications are discussed.

### Biosynthesis of MtNPs by probiotic bacteria and their application in agriculture

The use of probiotic microorganisms to produce MtNPs is an environmentally friendly as well as commercially attractive approach [47]. This is due to lower energy input, environmental sustainability, low costs, scalability and stability of MtNPs compared to the use of chemical synthesis methods. The non-pathogenicity of probiotics and their capacity to grow rapidly, regulating the expression of genes to produce various proteins and enzymes involved in the production of MtNPs is useful in many ways. *Lactobacillus* and *Bifidobacterium* are the most popular probiotics found in dairy products and natural flora in various parts of the body. These non-pathogenic gram-positive bacteria can be used in the production of a wide range of products [48]. The green synthesis of MtNPs, metal oxide nanoparticles (MONPs) and non-MtNPs by probiotics has been studied [49]. Probiotics exert their beneficial effects in a variety of ways, including direct effects on living cells and indirect effects on a wide range of metabolites. Probiotics have a negative electrokinetic potential that freely attracts cations, similar to other bacteria, which can be the starting point for the NP biosynthesis process [50].

The negative surface electrokinetic potential of *Lactobacilli* causes the rapid absorption of cations, which in turn plays a key role in the biosynthesis of MtNPs. Previous studies have reported biological adsorption and reduction of silver iodide by *Lactobacillus* sp. A09 [51] The tendency of *lactobacilli* to grow even in the presence of oxygen makes them metabolically highly viable. The bacterial redox potential decreases with the addition of reducing agents such as glucose. The oxidation–reduction potential represents the quantitative state of the degree of aerobiosis with the unit defined as rH2 (negative logarithm of the partial pressure of hydrogen gas). By adjusting the redox potential in the culture medium, the conditions can be changed in the desired direction. For example, suitable conditions can be created by lowering the rH2 for anaerobic conditions in the presence of oxygen, or by increasing the pH of the medium for creating aerobic conditions in an anaerobic environment. In this way, changing the different conditions of the culture medium plays an important role in the biosynthesis of MtNPs and/or MONPs. Various factors such as energy efficiency, glucose (which controls the value of rH2), ionic mean, pH, and total oxidation capacity (rH2) play an important role in the synthesis of NPs by *Lactobacillus* strains. Although *Lactobacilli* have a relatively weak metal detoxification system, a slightly acidic pH and a decrease in rH2 activates membrane-bound oxidoreductases and the metabolic pathway involved in MtONPs synthesis [52].

**Fig. 2:**
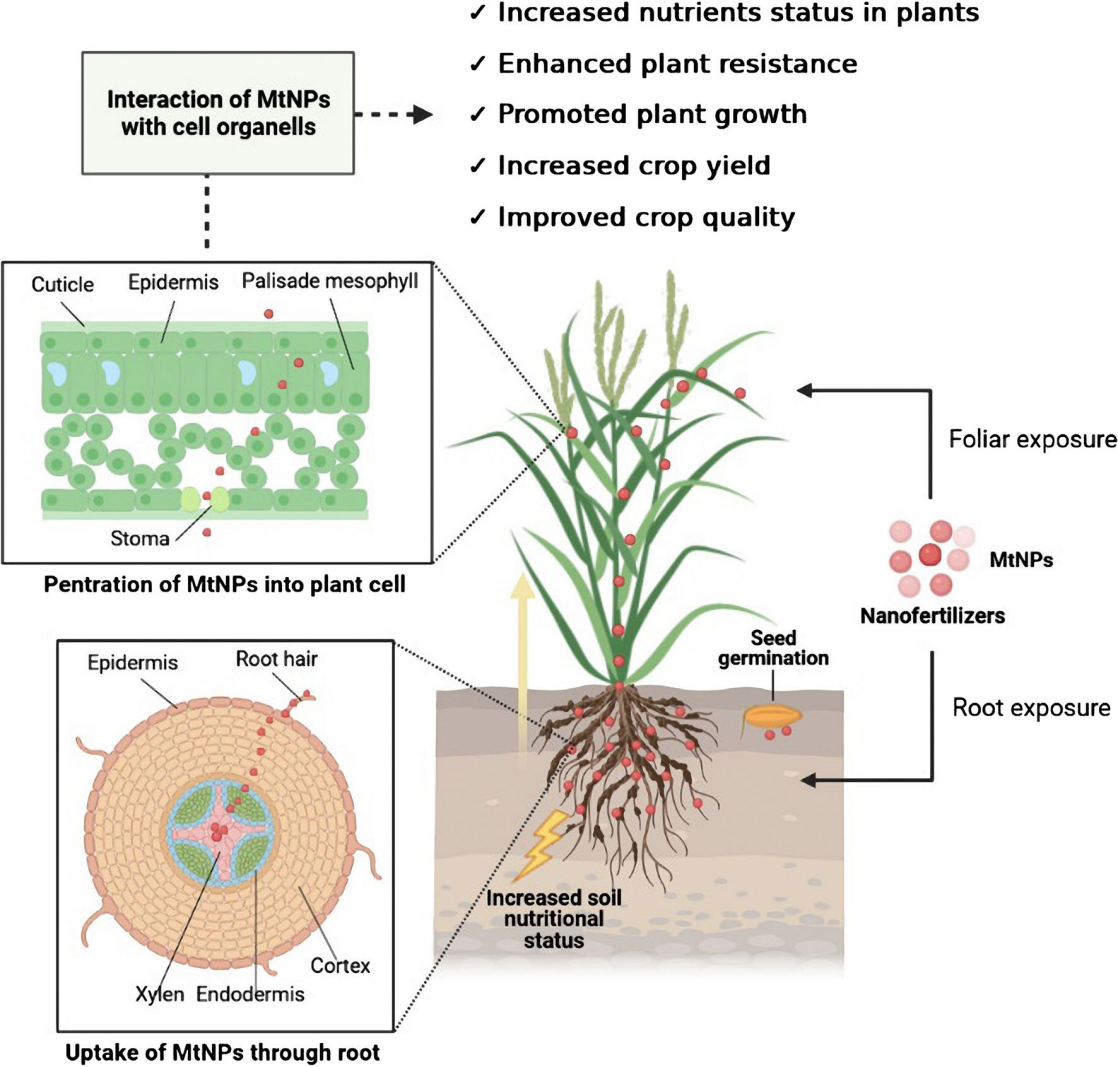
Schematic representation of the entry of MtNPs into plants through soil and roots or through extra-soil parts of plants as nanofertilizers and their uptake, translocation and potential effects on plants

MtNPs such as silver, gold, cadmium, copper, zinc, iron and selenium have applications in agriculture such as plant growth stimulation, antimicrobial and antifungal effects, nanofertilizers, nanobiosensors, plant micronutrients and plant disease control [53]. Table 1 shows a collection of probiotic species used for the synthesis of different MtNPs and their potential application in agriculture. Silver NPs (AgNPs) are amongst the most studied in biological systems and their various inhibitory and antimicrobial effects have long been known [54]. Various probiotics including gram-positive bacteria such as lactic acid bacteria, *bacillus*, *Staphylococcus*, *Brevibacterium* and gram-negative sp. Including *Pseudomonas* and *E*. *coli*, used for AgNP production. *Lactobacillus* sp. have been studied significantly as potential systems for AgNP production and Sásková and colleagues have demonstrated high extracellular production of AgNPs from silver ions by *Lactobacillus* *casei* sp. [55]. Similarly AgNP synthesis by *Lactobacillus* *acidophilus* have been shown to provide capping and reducing activities [56]. Gold NPs (AuNPs) are widely used in agriculture as antifungal and antibacterial agents and as delivery vehicles of fertilizer and pesticide sensors. The use of probiotics in the synthesis of AgNPs and AuNPs also eliminates the use of toxic chemicals and solvents, thus following the principles of green chemistry [57]. Cadmium sulfide (CdS) NPs are used in a wide variety of approaches such as biological sensors that have applications in medicine as well as in agriculture [58]. CdSNPs for use as nanosensors can be synthesized by probiotic bacteria. Nanosensors are useful in pesticide residue detection and can also detect soil moisture and soil nutrient levels [58, 59]. Copper is an essential micronutrient that is combined with many proteins and metalloenzymes and have a substantial role in plant metabolism and nutrition. CuNPs also have higher performance than bulk copper particles due to properties such as very small size and high surface-to-volume ratio compared to materials made from larger particles. The antifungal and antibacterial activity of CuNPs against gram-positive and gram-negative bacteria and pathogenic fungi has given them many applications in health and agriculture [60]. CuNPs have antifungal activity against plant pathogenic fungi such as *Fusarium* *oxysporum*, *Fusarium* *culmorum*, *Fusarium* *graminearum* *and* *Phytophthora* *infestans* [61]. They have also been reported to act as germinators and growth stimulants in some plants at concentrations below 100 ppm. So far, various chemical, physical and green synthesis methods have been used to synthesize CuNPs with different amounts, shapes and morphologies. Kouhkan et al. [62] reported that *Lactobacillus* *casei* is a promising source for the biosynthesis of CuNPs. Selenium is essential for the functions of most living organisms and is found in soil, water, seeds, livestock and food. Since SeNPs improve the plant’s ability to inhibit pathogens and activate antifungal properties, it is necessary to modify the Se content in plant nutrients by adding Se fertilizer to the soil and to balance Se in food [63]. Se-balanced food processing technology is a rapid process which helps to solve the Se imbalance issue in agriculture. Standardization of Se concentration in soil is very important and to achieve this pure Se compounds are used as fertilizer [64]. However, Se fertilizers remain in fertile topsoil during only one or few harvests and over a short period inorganic Se compounds are washed away by rain into the infertile horizons below the soil. Although the organic Se compounds are not actively leached, they are degraded quickly after applying. The advantage of SeNPs as nanofertilizers is that they do not leach slowly from the soil and do not dissolve in water or aqueous solutions [65, 66]. Figure 2 shows the potential effect of MtNPs as nanofertilizers on plants. Several different methods for synthesizing SeNPs have been described including synthesis of SeNPs using various probiotics including *Lactobacillus* *acidophilus*, *Lactobacillus* *casei* and *Bifidobacterium* sp. The shape, size, and quality of NPs produced by these probiotics differ from those generated by other methods. SeNPs produced by probiotics have a homogeneous particle size distribution and regular spherical shape [65, 67, 68].

**Table 1:** Nanoparticles synthesized by probiotic bacteria and their applications in agriculture

Probiotics	NPs	Production	NP size (nm)	Application in agriculture	Refs
*Lactobacillus*. *casei* ssp. *casei* *CCM* *7088*	Ag	Extracellular	12–27	Plant-growth stimulator, antimicrobial effect, antifungal effect	[53]
*L*. *acidophilus*	Ag	Extracellular	4–40	–	[54]
*Pseudomonas* *stutzeri*	Ag	Intracellular	Up to 200	Plant-growth stimulator, antimicrobial effect, antifungal effect	[65]
*Staphylococcus* *aureus*	Ag	Extracellular	160–180	Plant-growth stimulator, antimicrobial effect, antifungal effect	[83]
*Brevibacterium* *casei*	Ag	Extracellular	10–50	–	[188]
*Escherichia* *coli*	Ag	Extracellular	100	Plant-growth stimulator, antimicrobial effect, antifungal effect	[189]
*Bacillus* *cereus* *SZT1*	Ag	Extracellular	4 and 5	–	[190]
*Bacillus* *licheniformis* *Dahb1*	Ag	Extracellular	18.69–63.42	Antifungal effect	[191]
*Lactobacillus* *fermentum*	Ag	Extracellular	11.2	–	[192]
		Intracellular	15–40	–	
		Intracellular	60–80	–	
*Lactobacillus* *plantarum*	Ag	Extracellular	19.92 ± 3.4	–	[193]
*Lactobacillus* *rhamnosus*	Ag	Extracellular	233	–	[194]
*L*. *acidophilus* *58p*	Ag	Extracellular	30.65 ± 5.81	–	[193]
*Lactobacillus* sp. from *Yoghurt* *cells*	Ag	Extracellular	15–25	–	[237]
*L*. *delbrueckii* *isolated* *from* *probiotic* *curd*	Ag	Extracellular	54.3–112.7	–	[195]
*Actinobacter* spp.	Au	Intracellular	5–500	Antimicrobial effect, antifungal effect, nano fertilizer	[196]
*Bacillus* *subtilis*	Au	Extracellular	80 ± 0.18	–	[197]
*Escherichia* *coli* *k12*	Au	Extracellular	50	–	[70]
*L*. *casei* *(strain* *JCM1134)*	Au	Intracellular	ca.29.6	–	[198]
*Lactobacillus* *kimchicus* *DCY51T* *isolated* *from* *Korean* *kimchi*	Au	Intracellular	5–30	–	[57]
*Lactobacillus* *acidophilus* *DSMZ* *20079T*	CdS	Extracellular	2.5–5.5	Nanobiosensors	[58]
*Escherichia* *coli* *ATCC* *8739* *Bacillus* *subtilis* *ATCC* *6633*					
*Lactobacillus* *casei*	Copper	Extracellular	30–75	Plant micronutrient	[62]
*Lactobacillus* *acidophilus* *Lactobacillus* *casei* *Bifidobacterium* sp.	Se	Extracellular	50–500 50–500 400–500	Plant disease enhancer Nanofertilizer Nanofertilizer	[68]

### Biosynthesis of MtNPs by non-probiotics bacteria and their application in agriculture

Due to the growing need to develop new environmentally friendly technologies, the synthesis of MtNPs has received much attention as an advanced technology. Green synthesis of MtNPs by bacteria has become very important due to their relative ease of growth and lower production costs. Biosynthesis of AuNPs in three forms of spherical, triangular, and irregular (approximate size of 43.75 nm) has been reported using *Deinococcus* *radiodurans* [69]. In one study extracellular biosynthesis of AuNPs at room temperature using *Escherichia* *coli* *K12*. Generated a product that could reduce the toxic substance 4-nitrophenol in the presence of NaBH_4_ [70]. During the process of reducing 4-nitrophenol to 4-aminophenol, NaBH4 acts as a donor and prevents the formation of nitrophenolate (as a receptor). The rapid reduction of 4-nitrophenol to 4-aminophenol occurs when Ag/Au NPs are added to the reaction solution as a catalyst, which can be confirmed using the visible UV spectrum [71]. 4-Nitrophenol is a highly toxic organic compound and one of the most resistant contaminants in the effluents of various industries such as textile and dyeing. By spreading to the environment, this compound can contaminate soil and water leading to adverse effects on the central nervous system, liver and blood after ingestion of food grown in the contaminated areas. The development of a simple and effective method for the elimination or reduction of non-biodegradable bio pollutants into non-hazardous products is one of the serious challenges in environmental studies and agricultural systems. The product of chemical reduction of 4-nitrophenol is a useful and important compound called 4-aminophenol, which does not pose the risks of toxicity of 4-nitrophenol to the environment. The use of environmentally friendly green synthesis for produce nanoparticles as low-cost catalysts is a convenient method to chemically reduce toxic dyes such as 4-nitrophenol. MtNPs derive their catalytic capacity from their high surface-to-volume ratio. Due to their high adsorption level, MtNPs can provide conditions that increase the adsorption of the reactants on their surface and thus increase the reaction rate and reduce the activation energy level [72]. An *Acinetobacter* sp. species was able to synthesize AuNPs at 37 °C, pH 7, when treated with tetra-chloroauric acid (HAuCl_4_). These AuNPs were monodisperse or spherical and had antioxidant activity [73]. In a study of the biosynthesis of AuNPs using *Acinetobacter* sp. SW30 addition of HAuCl_4_ resulted in the biosynthesis of 10 to 20 nm polyhedral AuNPs. As the pH was increased to 9 and the temperature increased to 50 °C, more AuNPs were released into the solution [74]. *Acinetobacter* sp. SW30 has also been used at 30 °C and pH 7 to produce AuNPs with a monodisperse spherical shape and size of approximately 19 nm [75]. Reports indicate that filamentous *cyanobacteria* can biosynthesize AuNPs structures in various shapes, such as cubic, spherical, and octagonal, from the complexes of Au^+^-S_2_O^−2^_3_ and Au^3+^-NaCl [76, 77]. A *Cyanothece* sp. was able to synthesis AuNPs in the size range of 80 to 129 nm [78]. The first step in the interaction of *cyanobacterium* with Au3^+^ aqueous Cl^−^ is the deposition of NP sulfur Au^+^ on the cell wall and in the next step octagonal platelets forms of Au3^+^ are formed in solutions close to cell surfaces [77]. *Plectonema* *boryanum* UTEX 485, in the presence of S_2_O_3,_ was able to biosynthesize cubic form (sizes ranged from 10 to 25 nm) AuNPs in membrane vesicles. These bacteria also precipitated AuNPs in the form of octahedral platelets when incubated with AuCl_4_^−^ [76]. Electron transfer in the process of photosynthesis affects the biosynthesis of AuNPs in *cyanobacterium* cell wall. Cell membrane compositions in *cyanobacteria* can produce AuNPs by affecting the re-accumulation of gold in the cell wall. In general, at neutral pH, the biosynthesis of AuNPs takes place mostly in the periplasmic region of cyanobacteria. As the pH becomes more acidic, the more the synthesized AuNPs show different sizes and morphologies. Small AuNPs are deposited on bacterial cell walls at pH 2.0, while larger particles could be observed in the extracellular matrix. In general, changes in solution pH are a very influential factor in appearance and structure, as well as deposition location (extracellular or intracellular) of AuNPs [79]. Extracellular AgNP biosynthesis was demonstrated using *Pseudomonas* DC5 and *Pseudomonas* CA 417 [11]. In one study, the specificity of metal ion accumulation in the biosynthesis of AgNPs by *Pseudomonas* *stutzeri* AG259 was used to produce a range of shapes and sizes [80]. In one study, *Acinetobacter* sp. GWRFH45 biosynthesized AgNps [81]. Rapid biosynthesis of AgNps by *Enterobacteriaceae* has also been reported [82]. The reduction of Ag^+^ ions in *Staphylococcus* *aureus* led to the biosynthesis of AgNPs [83]. The use of bacterial cell culture supernatant to generate AgNPs of various shapes and sizes has been reported in several other studies [84]. In general, in the AgNPs biosynthesis cycle, the presence of nitrate ions in the presence of NADPH-dependent nitrate reductase enzymes (for free electron transfer) reduces the bioavailability of silver ions and ultimately causes spherical biosynthesis of AgNPs [79]. Au–Ag bimetallic NPs produced by a *Deinococcus* *radiodurans* synthesis system with a size of 149.8 nm showed the ability to decompose toxic triphenylmethane dye malachite green (MG) and convert it to the less toxic substance dimethylamino (benzophenone) [85]. The rapid and easy biosynthesis of a silver-gold double NPs functionalized with extremophilic *Deinococcus* *radiodurans* proteins (Drp-Au-AgNPs) led to the development of an environmentally friendly method for reducing polyphenyl from wastewater [85]. The ability of functionalized Drp-Au–Ag bimetallic MtNPs to degrade and reduce malachite green is attributed to a redox reaction as well as the alkaline conditions that amplify the electrostatic force between the functionalized Drp-Au–Ag bimetallic MtNPs and the malachite green molecules. Malachite green is a group of polyphenolic chemical dyes that are widely used in fishponds to repel pests and insects. Malachite green effluents, if released into the environment, in addition to proven mutagenic and carcinogenic effects in humans, can cause permanent dangerous and toxic effects. Nevertheless, the low price of green malachite is still a tempting factor to use this compound, so it can be considered an environmental problem. Although physical and chemical methods are used to remove polyphenyl compounds, the ability of nanoparticles as potential catalysts to absorb and then degrade polyphenol dyes is an efficient and environmentally friendly method for remediation [86]. In fact, nanobioremediation, is a new and efficient approach to clean up and remove contaminants and toxic compounds from the environment.

Extracellular biosynthesis of CdSNPs has been reported using *Klebsiella* *aerogenes*. The MtNPs ranged in diameter from 20 to 100 nm and their formation was highly dependent on the composition of the culture medium [87]. With the photosynthetic bacterium *Rhodopseudomonas* *palustris*, the extracellular biosynthesis of CdSNPs of approximately about 8 nm in diameter was dependent on cell growth stage and utilized the cysteine desulfhydrase located in the cytoplasmic space to stabilize the CdSNPs [88]. The results of a study on an intracellular CdSNP biosynthesized by *E*. *coli* showed that changes in growth phases affect the rate of biosynthesis and the size of CdSNPs. The biosynthesis rate of CdSNPs with a diameter of 2 to 5 nm in the stationary phase of *E*. *coli* was about 20 times higher than found in the logarithmic phase [89]. Extracellular biosynthesis of spherical CuNPs of 5–50 nm in size by *Streptomyces* *griseus* and 3.6–59 nm in size in e*ndophytic* *actinomycetes* *has* *been* *reported* [90]. A new species of *Desulfuromonas* *palmitatis* SDBY1 converts polycarbonate organic compounds to oxidized form in the presence of F^3+^, because F^3+^ can play the role of H_2_ receptor and be reduced [91]. Iron-reducing bacteria need electron-donating compounds during extracellular deposition of magnetite [92]. *Shewanella* *oneidensis* was used for the biosynthesis of Fe^2+^ and Fe^3+^ as extracellular magnetite. FeCl_2_, along with other salts, was used to reduce Fe^2+^ and Fe^3+^. The reduction of Fe^2+^ and Fe^3+^ seems to be facilitated by the transfer of salts by electron donation [93].

Although bacteria, viruses, and fungi are used to produce nanobiosensors with different MtNPs, nanoparticles produced of bacterial origin are mostly used as nanobiosensors in agricultural systems due to advantages such as production control, lower cost and high quality [94]. Bacterial NP-based biosensors, such as nanowires, nanoparticles and nanocapsule substrates are used specifically to diagnose plant diseases and are also used in cleaning strategies related to the accumulation of pesticides and insecticides in the food sector. Quantitative detection of insecticides containing dangerous and prohibited compounds such as organophosphorus, carbamate compounds is also done using biosensors [19]. In a study on a SeNP-based agricultural sensor to detect heavy metal toxicity, *Stenotrophomonas* *acidaminiphila* was used for SeNPs biosynthesis. This study presented a colorimetric method for the detection of heavy metals during bioremediation. In the absence of heavy metals, this process takes place naturally and the color changes to red, but in the presence of toxic heavy metals the process of selenium green synthesis to SeNPs is inhibited and the color changes. This synthesis is dependent on NADH reductase and increasing the concentration of toxic heavy metals causes a gradual decrease in enzyme activity and discoloration [95].

**Fig. 3:**
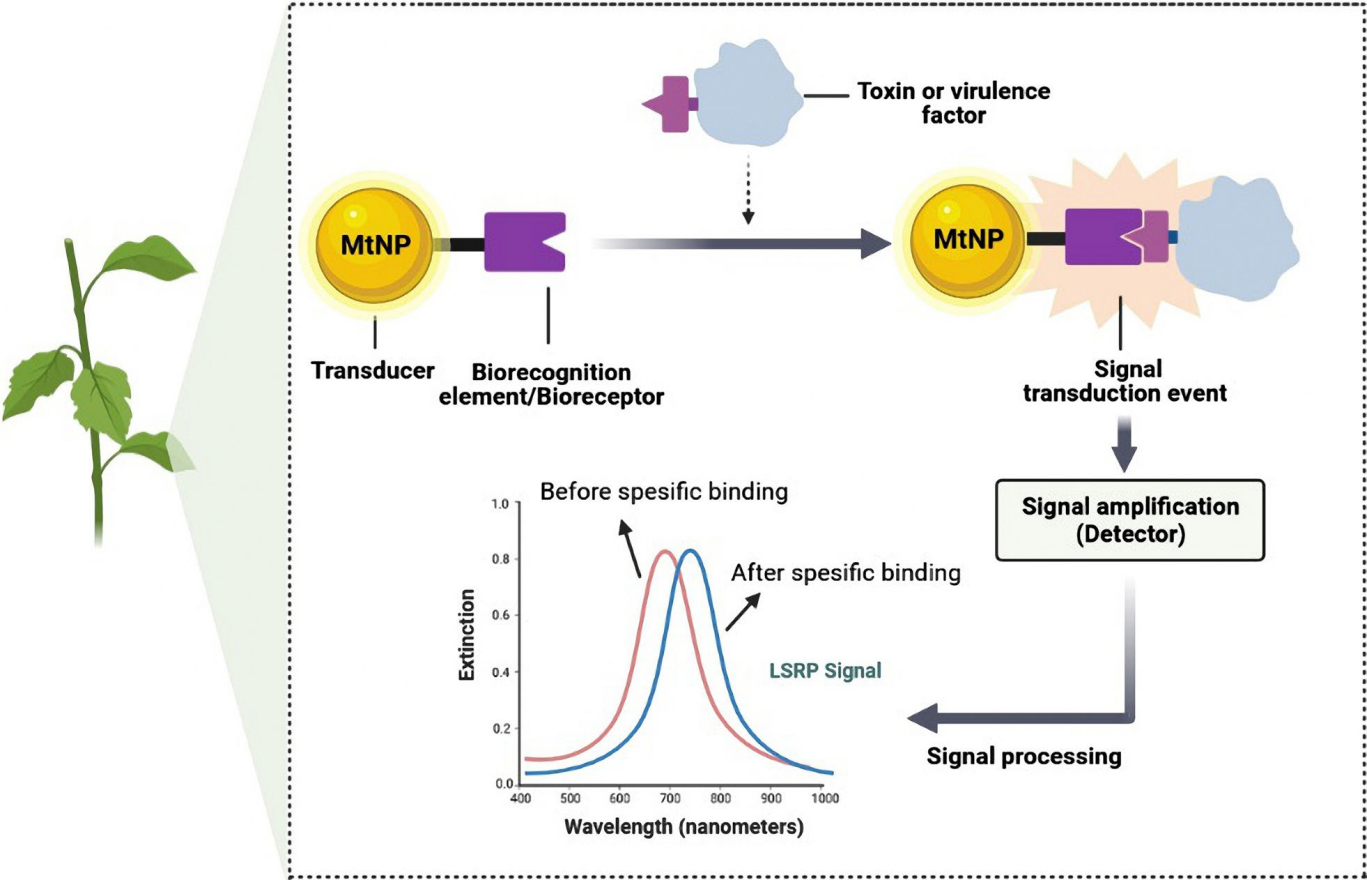
Schematic representation of the main constituents and working principle of MtNP-based biosensors for detection of plant pathogens and toxins

Several studies have examined the importance of using NPs as a diagnostic tool to identify a wide range of pathogenic bacteria in plants [96]. The application of nanoparticles in new technologies used in non-laboratory rapid screening methods for the detection of plant pathogens has a significant impact on the quality of agricultural products. In a study by Panferov et al. [97], an enhanced and rapid method based on lateral flow immunoassay (LFIA) was developed to detect low levels of potato leaf roll virus (PLRV) in contaminated fields. In this method, AuNPs were used as labels and silver ions were reduced at the AuNP surface to increase sensitivity [97]. In another report, infection of potato tubers with *Ralstonia* *solanacearum* was detected using an AuNP-based immunoassay. In this study, enhanced AuNP biosynthesized approach was used to increase sensitivity in lateral flow immunoassay (LFIA). The special feature of this method was a significant reduction in time to diagnose the cause of the infection [98]. In another study, the diagnosis of *Phytophthora* *infestans*, the causative agent of late blight in potatoes and tomatoes was performed using a combination of AuNPs-based lateral stream biosensor and asymmetric PCR to amplify the portion of the *Ph*. *infestans* genome. This showed that rapid detection of Phytophthora infestans in the early stages of infection can lead to appropriate management decisions to prevent the progression and spread of infection [99]. In another report, a rapid and inexpensive biosensing method was developed to identify the tomato yellow leaf ring virus genome using a AuNP-based probe and the local surface plasmon resonance (LSPR) method. Color changes were detected by UV–Vis spectroscopy, which indicates the presence of viral infection in the sample, eliminating the need for PCR and ELISA-dependent methods [100]. Although there are reports of successful use of MtNPs synthesized by non-biological methods in agricultural-related nanosensors, the importance of environmental protection has given priority to the development of methods for green MtNPs synthesis. The working principles of MtNP-based sensors for the detection of plant pathogens and toxins shown in Fig. 3.

Bacterial-synthesized NPs such as AgNPs have shown remarkable antibacterial effects and their application increases crop productivity, reduces waste generation, and saves energy and water when compared with common pesticides [37]. AgNPs are well-known antibacterial agents that can penetrate the bacterial cell wall and change the structure of the cell membrane by continuously releasing silver ions. Accumulation of AgNPs after anchoring to the cell surface can cause denaturation of the cell membrane. The binding of AgNPs to the cell wall increases the permeability of the cytoplasmic membrane and affects bacterial cell wall cross-linkage. With the entry of free silver ions into the cell, inactivation of respiratory enzymes occurs and the production of reactive oxygen species (ROS) increases, which causes damage to DNA and intracellular macromolecules and disrupts the cell membrane. AgNPs interrupts the electron transport chain and thus disrupts the production of adenosine triphosphate. In addition, the affinity of AgNPs to sulfur and phosphorus in the DNA structure causes serious damage to the DNA replication process, which in turn results in impaired cell reproduction. AgNPs directly disrupt protein production in the cytoplasm by denaturing ribosomes and also indirectly affect the natural structure of the proteins by increasing ROS levels, which together can lead to bacterial cell death. In general, many nanoparticles induce their antimicrobial effect by similar mechanisms [101]. However, despite the specific properties of each MtNP, most nanoparticles due to their general properties include antibacterial activity, disruption of the cytoplasmic membrane and cell wall, disruption of the energy transfer chain and electron transfer chain, toxic ROS production or DNA/protein oxidation, and Inhibition of enzymes makes their use in fungicides and pesticides important. For example, AuNPs in addition to accumulation at cell surface can exert its antimicrobial effect on the bacterial cell wall through electrostatic interactions [102]. The positive feature of using bio-pesticides is that they do not have the environmental disadvantages of using synthetic pesticides, but their effect on pests compared to the chemical pesticides is slow and limited [103]. Encapsulation of antimicrobial polypeptides may help to the endocytosis of these polypeptides surrounded by MtNPS. In addition to inducing cell death in pests such as insects, herbs and fungi MtNPs also can help in the controlled release of polypeptides into cells [104]. This has the added benefit of providing an important strategy in protecting the environment by reducing the dispersion of nanopesticides while encapsulation of medicinal plant repellents in MtNPs increases controlled release and reduces the level of toxicity of synthetic pesticides [105]. As a result of these features, nanobiopesticides can overcome the limitations of synthetic pesticides and biopesticides. With the use of nanoparticles, the active ingredients can be stabilized and made available through sustained-released giving effective and sustainable management for a long time without the hazards of using synthetic chemicals [106].

Several reports have evaluated the successful use of biological nanoparticles against pests. In one such study, spherical AuNPs and AgNPs biosynthesized from *Haloferax* *volcanii* were successfully used for antibacterial applications against two gram-negative bacteria [107]. Extracellular biosynthesis of AgNPs with high antimicrobial properties has also been reported using *Sporosarcina* *koreensis* DC4 [108]. The antifungal activity against *Fusarium* *graminearum* of an AgNPs biosynthesized by *Endophytic* bacteria has also been reported. In one study, biosynthesis of AgNPs was performed using *Pseudomonas* *poae* strain CO, in which the AgNPs with a diameter of approximately 20–50 nm showed antifungal activity [109]. Successful biosynthesis of AgNPs was reported in three strains of *Endophytic* *Streptomyces* spp. The biosynthesized NPs were spherical in shape, varying in size from at least 11 to a maximum of 63 nm, and acted against a wide range of single-celled fungi [110]. AgNPs (20 to 100 nm) biosynthesized using *Pseudomonas* *rhodesiae* culture medium supernatant showed strong antibacterial activity against *Dickeya* *dadantii* infection in sweet potato roots [111]. A haloalkaliphilic bacterium Streptomyces sp. was able to biosynthesize spherical AgNPs (diameter 16 nm) with high fungicidal properties against *Fusarium* *verticillioides*, one of the main causes of infection in cornfields by inhibiting ergosterol biosynthesis leading to inhibition of conidia germination and destruction of the *F*. *verticillioides* membrane [112].

CuNPs biosynthesized by an actinomycetes sp. isolated from *Convolvulus* *arvensis* also showed significant antifungal and antibacterial activity [113]. In one study, the effect of foliar application of different concentrations of CuNPs on the accumulation of bioactive compounds and antioxidant capacity in tomato fruits was estimated. CuNPs reduced the formation of ROS by increasing the activity of superoxide dismutase and catalase enzymes. In addition, the content of vitamin C, lycopene and phenol was increased in the presence of CuNPs. The results of this study also showed that CuNPs increased the strength of tomato fruits [114]. To investigate the effect of CuNPs biosynthesized by *Streptomyces* *griseus* on fungi that cause red root rot disease, experiments were performed on infected tea plantations. Comparison of tea plants treated with the chemical fungicide carbendazim, biosynthesized CuNPs or bulk copper showed that fungal resistance and leaf yield were higher in tea plants treated with biosynthesized CuNPs than in tea plants treated with carbendazim or bulk copper. Soil nutrients were also increased after the use of CuNPs. This study suggests that these CuNPs can be used as fungicides in the formulation of nanobiofertilizers [46, 90].

Several studies have examined the effect of MtNP size on their toxicity. Although factors such as size, concentration and zeta potential of MtNPs show various effects on different plants, there is a significant relationship between the size of MtNPs and the degree of toxicity created for the plant with the larger MtNPs being less toxic to plants than smaller ones. In addition, studies have shown that the concentration of nanoparticles also has a significant effect on their toxicity, for example, a concentration of more than 0.2 mg/ml CuNPs impairs plant growth and physiology [40].

The various MtNPs synthesized by non-probiotic bacteria with their potential applications in agriculture are summarized in Table 2.

**Table 2:** Non-probiotic resources for the biosynthesis nanoparticles and their applications in agriculture

Non-probiotics	NPs	Shape and location	Size (nm)	Applications in agriculture	Refs.
*Haloferax* *volcanii*	Au	Spherical Extracellular	10	Antibacterial activity Nanobiosensors	[107]
*Deinococcus* *radiodurans*	Au	Spherical, triangular and irregular Extracellular	43.75	Antibacterial activity	[69]
*Deinococcus* *radiodurans*	Au	-	149.8	Environmental remediation	[85]
*Escherichia* *coli* K12	Au	Highly dispersed Membrane	50	Environmental remediation	[70]
*Acinetobacter* sp. GWRVA25	Au	Monodispersed and spherical Extracellular	15 ± 10	Antioxidant activity	[73]
*Acinetobacter* sp. SW30	Au	Polyhedral Intracellular	20 ± 10	Environmental remediation	[74]
*Acinetobacter* sp. SW30	Au	Monodispersed spherical and polyhedral Intracellular	~ 19 to ~ 39	–	[75]
*Acinetobacter* sp. SW30	Au	Spherical Extracellular	10 ± 2	–	[199]
*Plectonemaboryanum* UTEX 485	Au	Cubic [Au (S_2_O_3_)_2_^3−^] and Octahedral [AuCl_4_^−^] Membrane vesicles	10–25	–	[76]
*Pseudomonas* *deceptionensis* DC5	Ag	Spherical Extracellular	–	Antimicrobial activity and biofilm inhibition activity	[11]
*Pseudomonas* *fluorescens* CA 417	Ag	Polydisperse	5–50 (TEM method) and 20.66 (DLS method)	Antibacterial activity against Nanobiopesticide feature	[200]
*Pseudomonas* *stutzeri* AG259	Ag	Equilateral triangles and hexagons Periplasmic	200	Biocide and antimicrobial agent	[80]
*Sporosarcina* *koreensis* DC4	Ag	Spherical	varied	Antibacterial activity	[108]
*Acinetobacter sp. *GWRFH 45	Ag	Monodispersed spherical Extracellular	10 ± 5	Antifungal and biofilm inhibition	[81]
*Staphylococcus* *aureus*	Ag	-	10–15	Antibacterial activity	[83]
*Pseudomonas* *rhodesiae*	Ag	Spherical Extracellular	20–100	Antibacterial activity	[111]
*Pseudomonas* *poae* *CO*	Ag	Spherical Extracellular	19.8–44.9	Antifungal activity	[109]
*Streptomyces* *capillispiralis* *Ca-1* *Streptomyces* *zaomyceticus Oc-5* *Streptomyces* *pseudogriseolus* *Acv-11*	Ag	Spherical Extracellular	23.77–63.14 11.32–36.72 11.70–44.73	Antibacterial activity Antifungal activity Biocatalysts Larvicidal	[110]
*Haloalkaliphilic* *Streptomyces *spp.	Ag	Spherical Extracellular	16.4 ± 2.2	Antibacterial activity Antifungal activity	[112]
*Klebsiella aerogenes*	CdS	Extracellular	20–200	Antibacterial activity	[87]
*Rhodopseudomonas* *palustris*	Cd	Face-centered cubic Extracellular	8.01 ± 0.25	Antibacterial activity	[88]
*E*. *coli*	Cd	Intracellular	2–5	–	[89]
*Streptomyces* *griseus*	Cu	Polydisperse Extracellular	5–50	Nanobiofungicides	[90]
*Endophytic* *actinomycetes* *Ca-1*	Cu	Spherical-monodispersed Extracellular	3.6–59	Nanobiopesticide	[113]
*Shewanella* *oneidensis*	Fe^2+^ Fe^3+^	Extracellular	–	Nanobiosensors Nanobiomarker	[93]

### Biosynthesis of MtNPs by Fungi and their application in agriculture

Nanotechnology touches many fields, including agriculture and plant disease management. In recent years, fungi have been added to the list of microorganisms used in the production of nanoparticles. Among the various microorganisms used to synthesize nanoparticles, fungi are effective candidates for making intracellular and extracellular MtNPs. Nanoparticles made using fungi have good dispersion and stability characteristics. The attractiveness of using fungi in the production of nanoparticles is due to the presence of significant amounts of specific enzymes in these microorganisms, ease of working with them in the laboratory, scalability and financially economic growth of fungi even on an industrial scale making myconanotechnology an environmentally friendly and cost-effective option [115, 116]. Although there are several methods for synthesizing MtNPs from fungi, little is currently known about potential drawbacks and limitations. Filamentous fungi can produce a wide range of MtNPs such as gold, silver, iron oxide, and even bimetallic nanoparticles [117, 118]. Research has shown that several different species of fungi can be used in the green synthesis MtNPs with the desired size, surface charge and morphology, and desirable properties including *Pestalotiopsis* sp., *Phoma* sp., *Humicola* sp., *Fusarium* *oxysporum,* *Aspergillus* *niger,* *Trichoderma* sp., *Hormoconis* *resinae,* *Phaenerochaete* *chrysosporium* and *Penicillium*. Using fungi as reducing and stabilizing agents for the biosynthesis of AgNPs has been considered due to their high efficiency, ease of operation and low residual toxicity. The mechanisms of synthesis are not yet fully understood, but synthesis can be optimized by adjusting parameters such as silver salt concentration, biomass, temperature, pH and fungal cultivation time. As with bacterial produced AgNPs, similar structures synthesized using fungi, with low toxicity and good biological compatibility, can control pathogens [40, 119].

These findings set the stage for future research into the use of these MtNPs as antimicrobials agent in agriculture sector. Among the various types of MtNPs studied to date, AgNPs stand out due to their wide range of antimicrobial potential [120–122]. These MtNPs attach to the cell wall and membrane of the microorganisms and may also enter the cell. They damage cellular structures, induce the production of ROS, and alter signal transduction mechanisms [123, 124]. The use of fungi for the synthesis of AgNPs involves culturing the fungus on agar and then transferring it to a liquid medium. The produced biomass is then transferred to water to release the compounds that act in the synthesis of MtNPs. After filtration, the biomass is discarded and silver nitrate is added to the filter [125, 126]. One of the first reports of the synthesis of AuNPs by fungi was shown by *Verticillium* sp. [127], though other fungi including *Penicillium* sp. *Hormoconis* *resinae,* *Candida* *albicans,* *Alternaria* *alternate*, *Paraconiothyrium* *variable,* *Aspergillus* sp., *Volvariella* *volvacea,* *Colletotrichum* sp. and *Trichothecium* sp. have also been used successfully for AuNP production. The living and dead cells of *Aspergillus* *oryzae* also produce AuNPs in a process that is economically viable for use in the food industry [128]. The fungus *Colletotrichum* sp, which has a parasitic life and grows on geraniums, produces AuNPs with rod-like and prism-like morphology when exposed to chlorate ions [129]. In addition to MtNPs, the production of Au–Ag bimetallic alloys is possible using *F*. *oxysporum*. In a recent study, it was shown that to exposure of *F*. *oxysporum* can stimulate accumulation of metal ions by physicochemical and biological mechanisms such as extracellular binding by polymers and metabolites, binding to specific polypeptides, and metabolism-dependent accumulation [130]. Exposure of *F*. *oxysporum* biomass to co-molar solutions of HAuCL_4_ and AgNO_3_ has also been shown to produce highly stable Au–Ag alloy nanoparticles with different molar ratios and it has been shown that NADH factors play a very important role in determining the chemical composition of Au–Ag alloy nanoparticles [129]. In addition, exposure of *F*. *oxysporum* to aqueous solution of CdSO_4_ causes extracellular production of CdSNPs. The particles produced by this method have a uniform dispersion and their dimensions are in the range of 5 to 20 nm [131]. Cadmium quantum dot nanoparticles are produced by using fungi such as *Coriolus* *versicolor*, *Schizosaccharomyces* *pombe*, *Candida* *glabrat* and *F*. *oxysporum* [115]. Other important applications of fungi include the production of zirconia nanoparticles with many applications. Reaction of the aqueous solution of k2ZrF6 with *F*. *oxysporum*, hydrolysis of zirconium hexafluoride anions occurs extracellularly and crystalline zirconia nanoparticles are produced at room temperature [132].

**Fig. 4:**
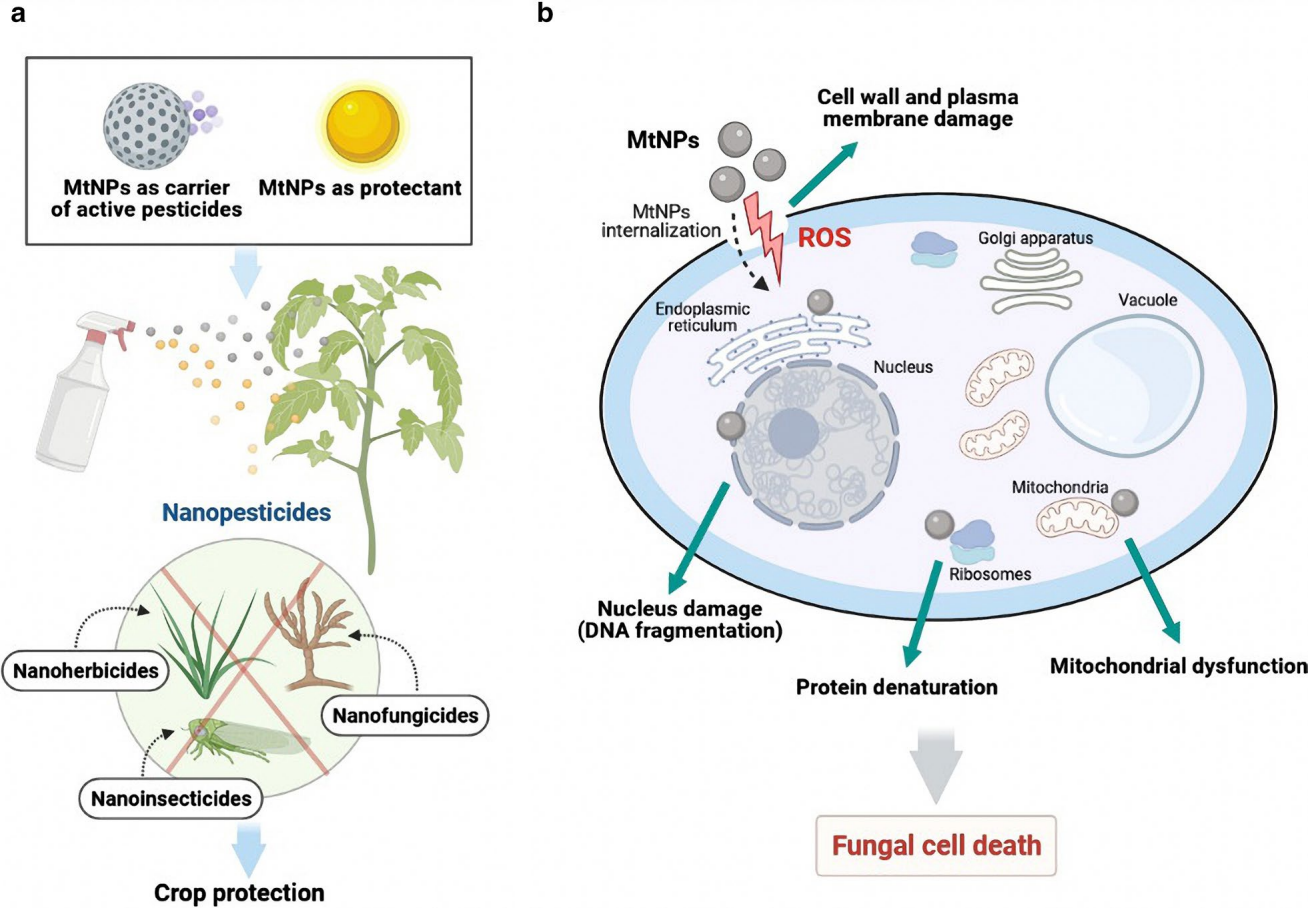
Application of MtNPs as nanopesticides: **a** MtNPs act as nanopesticides targeting a wide range of pests and phytopathogenic agents and as a carrier for pesticides to provide crop protection, **b** Mechanisms of action of MtNPs as nanofungicides. MtNPs act on the fungus cell wall, leading to membrane damage. Disruption of the membrane by MtNPs causes pore formation. After internalization, MtNPs target main cellular organs such as the nucleus, ribosomes and mitochondria, causing cell death

Myconanotechnology has established a new field of research in the production of antifungal nanoparticles. The antifungal properties of AgNPs against rose powdery mildew caused by S*phaerotheca* *pannos *var. *rosae* were have been demonstrated by spraying a large contaminated surface area with nanosilver solution. Two days later, more than 95% of the rose powder had been eliminated and no recurrence was observed for a week [133]. In a related study, AgNPs had a toxic effects on the pathogen *Colletotrichum* *gloesporioides*, which causes anthracnose in a several fruits showing significant growth retardation of the *C*. *gloesporioides*. As a result, AgNPs can be introduced as a fungicide for the management of plant diseases [134]. AgNPs were synthesized using *Epicoccum* *nigrum* and their antifungal activity was observed against pathogenic fungi such as *Fusarium* *solani,* *Sporothrix* *schenckii,* *C*. *albicans,* *Cryptococcus* *neoformans,* *Aspergillus* *flavus* and *Aspergillus* *fumigatus* *and* AgNPs were synthesized using *Guignardia* *mangiferae* were active against the phytopathogenic fungi *including* *Rhizoctonia* *solani*, *Colletotrichum* sp. and *Curvularia* *lunata* [135]. Antifungal effects of AgNPs synthesized by the plant pathogen *Fusarium* *solani* isolated from wheat showed activity against various other species of phytopathogenic fungi that cause diseases of wheat, barley and corn kernels [136]. MtNPs are active against a wide range of pests and their use in the formulation of pesticides is easily achieved [137, 138]. Porous hollow silica nanoparticles (PHSN) have been shown to be effective for controlled release of water-soluble pesticides and in improving their transport to target locations [139]. AgNPs synthesized using *Aspergillus* *versicolor* have been shown to be effective against infection with *Botrytis* *cinerea* and *Sclerotinia* *sclerotiorum* in strawberry plants [140]. Figure 4a shows the various MtNPs can act as either plant protectants against pests or as carriers of pesticides. Figure 4b shows the general mechanism of action of MtNPs as nanofungicide.

Nanoparticles produced by fungi have coatings that are obtained directly from the fungi and which make them more stable. Depending on the fungus used, the cap may have biological activity and a synergistic effect with the nanoparticle core. These attributes contribute to the efficacy of nanofertilizers in achieving slow secretion or secretion due to biological and physical activation. At the same time, nanofertilizers improve plant nutritional efficiency and prevent excessive toxicity of chemical fertilizers. Thus, it helps developing countries in particular in establishing sustainable agricultural programs [141].

However, while there are several strong advantages for using fungi for green synthesis of MtNPs, there are also drawbacks that need to be addressed. These include determining which fungus is best for producing nanoparticles with the desired properties, determining the appropriate parameters for growth, the need for sterile conditions as well as the time required for the fungus to grow, and completing its synthesis. There may also be problems with scale-up production, including the need to further investigate the mechanisms by which cap layers are formed and the molecules contained in them. While more research is needed, studies showed that using fungi for the green synthesis of MtNPs has the potential to address a wide range of possible applications especially for the control of pests [135]. A summary of some fungal sources for the production of MtNPs with specific characteristics and potential applications in agriculture is shown in Table 3.

**Table 3:** Potential fungal isolates used for the biosynthesis of nanoparticles and their applications in agriculture

Fungi	NPs	Shape and location	size (nm)	Applications in agriculture	Refd.
*Fusarium* *oxysporum*	CdS	Spherical Extracellular	5–20	Antibacterial activity	[131]
*Fusarium* *solani*	Ag	Spherical, extracellular	5–35	Textile fabrics, antifungal	[201]
*Fusarium* *culmorum*	Ag, Au, Pb, Cu	Spherical, extracellular	5–10	–	[202]
*Aspergillus* *oryzae* *var*. *viridis*	Au	Various shapes Mycelial surface	10–60	–	[128]
*Aspergillus* *niger*	Au	Nanowalls, spiral plates, polydispersed or spherical,	12.8–20	Toxic to mosquito larvae	[203]
	Ag	Spherical, extracellular	3–30	Antibacterial and antifungal activity	[204]
*Aspergillus* *flavus*	Ag	Spherical, On cell wall surface	8.92–17	–	[136]
*Aspergillus* *clavitus*	Au	Triangular, spherical and hexagonal Extracellular	24.4 ± 11	–	[205]
	Ag	Extracellular	100–200	Antimicrobial activity	[206]
*Aspergillus* *terreus*	Ag	Spherical, extracellular	1–20	–	[207]
*Alternaria* *alternata*	Ag, Cd	Spherical, extracellular	20–60	Enhancement in antifungal activity of fluconazole against Phoma glomerata and water quality monitoring, antifungal combined with Fluconazol	[208]
	Au	Spherical, triangular, hexagonal Extracellular	12 ± 5	–	[209]
*Rhizopus* *stolonifer*	Au	Irregular Extracellular	1–5	–	[210]
	Ag	Quasi-spherical	25–30	–	[210]
*Rhizopus* *oryzae*	Au	Nanocrystalline Cell surface	10	Nanopesticides	[211]
*Phyllanthus* *amarus*	Ag	Spherical, extracellular	30	–	[212]
*Pleurotus* *sajor-caju*	Au, Ag	Spherical, extracellular	20–40	–	[213]
*Penicillium* *fellutanum*	Ag	Mostly spherical, Extracellular	5–25	–	[214]
*Penicillium* *strain* *J3*	Ag	Mostly spherical	10–100	–	[215]
*Penicillium* *brevicompactum* *WA2315*	Ag	Spherical, extracellular	58.35 ± 17.88	–	[216]
*Penicillium* *brevicompactum*	Au	Spherical, triangular and hexagonal Extracellular	10–60	–	[217]
*P*. *nagiovense* *AJ12*	Ag	Spherical Cell-free filtrate	25 ± 2.8	–	[218]
*P*. *rugulosum*	Au	Spherical, triangular, hexagonal	20–80	–	[219]
*Penicillium* sp. *1–208*	Au	Spherical Cell filtrate	30–50	–	[220]
*Trichoderma* *viride*	Ag	Mostly spherical	2–4	Biosensor and bio imaging	[238]
		Spherical, rod-like	5–40	Antibacterial activity Vegetable and fruit preservation	[239]
*Trichoderma* *asperellum*	Ag	Nanocrystalline or spherical Extracellular	13–18	–	[221]
*Trichoderma* *reesei*	Ag	Extracellular	5–50	–	[222]
*Trichoderma* *Koningii*	Au	Spheres Cell-free filtrate	10–40	–	[223]
*Trichoderma* *harzianum*	Cu, Ag	Spherical. Extracellular	20–35	Antifungal, Antiparasitic combined with Triclabendazol, Insecticide	[208]
*Volvariella* *volvaceae*	Au–Ag	Triangular, spherical, hexagonal Extracellular	20–150	–	[224]
*Cladosporium* *cladosporioides*	Ag	Mostly spherical or hexagonal Extracellular	10–100	–	[225]
*Cylindrocladium* *floridanum*	Au	Spherical Extracellular	19.5	–	[226]
*Cochliobolus* *lunatus*	Ag	Spherical. Extracellular	5–10	–	[227]
*Coriolus* *versicolor*	Ag	Spherical Intra- and extracellular	25–75, 444–491	–	[228]
	Au	Spherical and ellipsoidal Intra- and extracellular	20–100, 100–300	–	[240]
*Verticillium* sp.	Au	Spherical. Cell wall, cytoplasmic membrane and intracellular	20	–	[229]

### Biosynthesis of MtNPs by yeasts and their application in agriculture

Yeasts are the unicellular microorganisms that reproduce during an asymmetric cell division process called budding and can be categorized as Ascomycetes such as *Saccharomyces* and *Candida* or Basidiomycetes such as *Filobasidiella* and *Rhodotorula* [142]. In addition to traditionally use of yeasts for production of several fermented food such as alcoholic beverages and bakery products modern application of yeasts include the production of heterologous compounds, single cell protein (SCP) and their use in the biofuels industry [142]. Yeasts also play an important role in agriculture as biological control agents, biological treatments and as indicators of a quality environment [143]. They grow easily on low-cost media and can adapt to harsh environmental conditions such as a wide range of temperature and pH and high concentrated organic and inorganic pollutants. Yeasts have the inherent ability to absorb and accumulate large concentrations of toxic metal ions from the environment and can adapt themselves to this environmental stress using various detoxification mechanisms such as mobilization, immobilization or metals transformation. These bioremediation mechanism of yeasts can play key roles for the green synthesis of MtNPs [144]. The stress caused by the presence of metal ions leads to activate a metabolic cascade of chemical reactions for the synthesis of stress-relieving compounds such as phytochelatin synthase and glutathione that have redox and nucleophilic features. These compounds bind to metal ions such as cadmium, zinc, silver, selenium, gold, nickel, copper, etc. reduce them to the respective MtNPs. Additional mechanisms take in this process include the activity of membrane-bound oxidoreductases and quinones. Adsorption of metal ions leads to an increase in pH and subsequent activation of pH-sensitive oxidoreductases, which act as both reducing and stabilizing agents for MtNP synthesis. Depending on the yeast species type, the biosynthesis of MtNPs can either be intracellular or extracellular [145].

Many Yeast species such as *Saccharomyces* *cerevisiae*, *Saccharomyces* *boulardii*, *Candida* *utilis* *NCIM* *3469*, *Candida* *lusitaniae*, silver-tolerant yeast strain MKY3 and a marine yeast *Yarrowia* *lipolytica* strain have been used for the biosynthesis of AgNPs [25, 44]. In a recent study Elahian et al. [146] utilized a genetically modified strain of *Pichia* *pastoris* for AgNP biosynthesis. The yeast *Pichia* *jadinii* (formerly *Candida* *utilis*), isolated from a metal-rich dump, has been shown to produce AuNPs from the metal [147]. The green synthesis of AuNPs using the tropical yeast *Yarrowia* *lipolytica* is also described by Agnihotri et al. [148]. It has also been demonstrated that extremophilic yeasts, isolated from acid mine drainage, are able to produce AuNPs and AgNPs [147]. Biosynthesis of other MtNPs such as CuNPs and Palladium nanoparticles (PdNPs) using *Saccharomyces* *cerevisiae* have been also reported [149].

Fernandez et al. [150], demonstrated antifungal activity of AgNPs synthesized using two epiphytic yeasts, *Cryptococcus* *laurentii* and *Rhodotorula* *glutinis* isolated from apple peel and its potential application as an efficacious nanofungicide against phytopathogenic fungi that cause postharvest diseases in pome fruits has been reported. Because epiphytic yeasts, like *C*. *laurentii* and *R*. *glutinis*, are harmless and are regard as GRAS (Generally Recognized As Safe) microorganisms, MtNPs production using these two yeasts has significant advantages in the application of agroecosystems [151].

### Biosynthesis of MtNPs by microalgea and their application in agriculture

Microalgae, single-celled prokaryotic or eukaryotic predominantly aquatic microorganisms that undertake photosynthesis form colonies without any cell differentiation and can grow in a variety of environments, such as freshwater, saline, and sea, where their growth is directly related to temperature, light intensity, and nutrient concentration [152]. Microalgae have been widely used in a variety of industrial, health and biotechnological applications thanks to a wide range of potential biological applications, such as pigment overexpression, biological treatment, biofuel production and toxicity studies [153]. These photosynthetic microorganisms are very sensitive to environmental changes and can detect traces of contaminants, so they can be used as biosensors to detect contaminants such as herbicides, heavy metals and volatile organic compounds in the range of 1–10 ppb. Depending on their biological constituents, microalgae react selectively with some contaminants, which can result in electrical, thermal or optical signals which can be identified, processed and analyzed by microprocessors [154]. Microalgae-based synthesis of the MtNPs, known as "phyco-nanotechnology", is an emerging field with a wide range of potential applications [155]. Many phototrophic microorganisms belong to the microalgae, and can be used to produce secondary metabolites and substances with unique properties including carotenoids, enzymes, fatty acids, polymers, peptides, antioxidants, toxins and sterols [156].

Several reports have shown that some microalgae not only be able to accumulate heavy metals intracellularly or extracellularly, but they also have the ability to synthesize MtNPs such as silver, gold, cadmium and platinum [157]. In addition to the low cost of nanoparticles biosynthesis using microalgae, synthesis can also be performed at low temperatures with higher energy efficiency, lower toxicity and lower risk to the environment [158].

The mechanism of biosynthesis of MtNPs by microalgae is not yet well understood. However, it is clear that nanoparticles can be synthesized by extracellular and intracellular mechanisms from algal biomass. In the case of extracellular production the bioreduction of a metal ion MtNPs takes place on the surface of the microalgae cell whereas in the intracellular mechanism the process of enzymatic reduction takes place inside the cell [159]. It has been reported that intracellular polyphosphates and extracellular polysaccharides as well as carboxyl groups on the cell surface absorb metal ions through electrostatic interaction and then metal particles enter the cell and are captured during the processes used to form MtNPs [160]. Extracellular pathway synthesis of MtNPs by microalgae is carried out with the aim of eliminating the effects of toxic metals using reductase enzymes and shuttle quinones and by secreting extracellular enzymes or by electrostatic interactions between metal ions and cell surface constituents [160]. The synthesis of MtNPs also occurs through the activity of intracellular terpenoids, carbonyl groups, phenolic, flavonoids, amines, amides, proteins, pigments, alkaloids as reducing agents. Many methods have been described for synthesizing MtNPs from saline solutions using microalgae to improve the size, shape of nanoparticles and higher quality [161]. These include the use of biological molecules extracted from lysed microalgae cells, the use of cell-free supernatant, or the biological synthesis of nanoparticles from living microalgae. Several microalgae species have been used for the biological synthesis of MtNPs using their extracted biomolecules [160]. To obtain AuNPs, the algal biomass is first lyophilized and then reverse-phase-high performance liquid chromatography (RP-HPLC) carried on to purify the gold-shaped protein (GSP) which is responsible for guiding the shape of the nanoparticles. This protein is then placed in aqueous HAuCl_4_ solution for the synthesis of nanoparticles of different shapes. In the case of AgNPs low molecular weight proteins (PLW) and high molecular weight proteins (PHW) in algal biomass are responsible for reducing silver ions in their metallic type. *Spirogyra* *insignis* (Charophyta) fine powder is used for biosynthesis of both AgNPs and AuNPs [162]. AgNPs have also been synthesized using cell-free supernatants of cyanobacterium and chlorophyta cell lysates [160].

One of the problems of using microalgae in biosynthesis of MtNPs in bioreactors on an industrial scale is their precipitation in the culture medium. However, immobilization of microalgae in organic matrices (polyvinyl alcohol, polysulfone) and polymers matrices (alginate, carcinogen, chitosan and silica gel) is one of the solutions to this problem and recycling of microalgae [163]. Once stabilized in organic matrices, microalgae retain their ability to synthesize nanoparticles after which they are released into a matrix in a complex culture medium. Biosynthesis of AgNPs from different microalgae species such as chlorophyta, haptophyta and ocrofita has also been reported by different groups [164, 165]. A summary of reports of the biosynthesis of MtNPs by microalgae is presented in Table 4.

**Table 4:** Microalgae used for the biosynthesis of nanoparticles

Microalgae	NPs	Morphology mode of synthesis	Size (nm)	Refs.
*Chlorella* *vulgaris*	Au	Spherical Extracellular	2–10	[230]
*Chlorella* *vulgaris*	Ag	Triangular	28	[170]
*Chlorella* *pyrenoidosa*	Au	Icosahedral and spherical	25–30	[231]
*Chlamydomonas* *reinhardti*	Ag	Rectangular and round Extracellular	1–15	[232]
*Diatoms*	Au	–	–	[167]
*Klebsormidium* *flaccidum*	Au	Extracellular	10–20	[233]
*Tetraselmis* *kochinensis*	Au	Triangular, FCC, and spherical, Intracellular	5–35	[159]
*Pithophora* *oedogonia*	Ag	Cubical and hexagonal,	24–55	[234]
*Chlorococcum* *humicola*	Ag	Spherical	16	[235]
*Chlamydomonas* *reinhardtii*	Ag	Rectangular and rounded	1–15	[168]
*Enteromorpha* *flexuosa*	Ag	Circular	15	[236]

The synthesis of AgNPs by microalgae has great potential due to the high growth of algal microbiomes during the biosynthetic process and also the increase in the surface area of silver in the nanometer range [166]. AgNPs synthesized by microalgae may exhibit their antibacterial effect by altering the permeability of cell membranes and airways [167]. Antifungal activity of AgNPs by inhibiting the growth of fungal hyphae have been reported [168]. However, nanoparticles biosynthesized by microalgae show a greater inhibitory effect [169]. El-Moslamy et al. [170] showed the effective role of AgNPs synthesized by *Chlorella* *vulgaris* in controlling plant diseases with strong antifungal activity against *Alternaria* *alternata*, the causative agent of leaf spot disease and plant rot. AgNPs produced by the microalga *Chlorococcum* *humicola* with the help of microalgal biomass activity against *Candida* *albicans,* *Aspergillus* *niger* and *Aspergillus* *flavus* showed significant growth inhibition against *C*. *albicans*. Biomass containing *Chlorella* sp. and *Haematococcus* *Candida* *albicanspluvialis* inhibited the growth of *Penicillium* *expansum*, the main cause of loss of quality and quantity of fruit after harvest [152, 171].

## Challenges and future direction of using MtNPs in agriculture

Green synthesis of MtNPs using microorganisms is a promising and environmentally friendly approach for agricultural applications such as nanofertilizers, nanopesticides and nanobiosensors. Given their potential widespread use in the future it is likely that large volumes of MtNPs produced by different methods will enter ecosystems [172]. Despite the favorable physical and chemical properties of MtNPs, the complexity of soil-crop ecosystems means that the environmental behaviors of these nanoparticles are not yet fully predictable after use, and this remains an important challenge [173]. Therefore, before fully utilizing their potential, it is necessary to evaluate the effects and interaction with living systems. At this stage, screening of nanomaterials is essential to assess their potential toxicity and to understand their mechanisms of action to prevent their adverse effects in the future [174].

The nanoscale dimensions of MtNPs, which determines many of their beneficial properties, can potentially also increase their potential adverse effects [172]. The toxicity of MtNPs is influenced by various factors such as solubility and their binding specificity to biological sites [175]. Several studies have shown the unpleasant aspect of long-term exposure to some MtNPs such as AuNPs and AgNPs. In a study by Vecchio et al. [174] the in vivo toxicity of AuNPs in *Drosophila* *melanogaster* was evaluated. Due to the mutations that can be passed on to offspring, significant phenotypic changes were observed in later generations of Drosophila after treatment with AuNPs, indicating the potential severity of AuNP toxicity. These findings provide important evidence of the adverse effects of AuNPs on the growth and development of organisms. These studies also demonstrate the need for reliable evaluation of the toxicological properties of nanomaterials and the need for significant efforts by the nanoscience community to produce biocompatible nanomaterials without any adverse effects on human health and the environment [174].

AgNPs are primarily produced for antiseptic applications and have potential antimicrobial activity against many pathogenic microorganisms. However, together with this favorable feature, AgNPs also show impermissible toxic effects on human health and ecosystems. Ecologists have warned that if these nano-antimicrobials are released into the environment, their spread could have serious negative consequences for other microorganisms in natural ecosystems. There is ample evidence that AgNPs are not only toxic to bacteria, but also to the cells of other organisms such as brain cells, liver cells, and stem cells, which can lead to severe damage [175]. MtNPs cause toxicity through important cellular processes such as increased levels of ROS, decreased intracellular glutathione levels, and decreased mitochondrial membrane potential. AgNPs can adversely affect on cells and embryos of freshwater fish. In one study, the toxic effects of AgNPs on adult Japanese rice fish (*Medaka,* *Oryzias* *latipes*) were evaluated by exposure to these nanoparticles. The results showed a decrease in the activity of lactate dehydrogenase and antioxidant enzymes in the liver, glutathione depletion and lipid peroxidation in the liver and gills, with varying degrees of histological lesions in the tissues [176].

Several studies have shown that MtNPs can also have an adverse effects on key major elements (plant, soil and water) in agroecosystems [25]. Generally MtNPs can enter the agricultural ecosystem through both direct and indirect routes [173]. MtNPs used for agricultural applications can enter soil, climate, and atmosphere through washing, rainfall, airflow, and trophic transfer. Various studies have shown that these MtNPs may be absorbed by microorganisms in the soil, sediments and plant roots. These MtNPs are then transferred from the roots to other parts of the plants where they can accumulate [25]. Accumulation as a key behavior of MtNPs can significantly affect their fate and toxicity in the agricultural system [173]. Standardization of MtNPs use is therefore required for their safe and sustainable use in agriculture [25]. Biogenic MtNPs can be potentially toxic directly to plants, to plant-related beneficial microbes and eventually to human. Therefore, when using MtNPs directly in crops special attention must be paid to the interaction between nanoparticles and the treated plants [25, 172, 177]. The interaction between MtNPs and plants leads to numerous physiological, morphological and genotoxic changes that must be fully understood to ensure effective application of nanotechnology in agriculture. The effects of MtNPs on plants vary according to the growth stage of the plant, the time of exposure to nanoparticles, the adsorption method as well as the different physical and chemical properties of the plants themselves [178]. However, some MtNPs have a positive effect on the plant system and can improve seed germination and stimulate growth parameters, though these effects can differ between different plants [178]. Several studies have also reported significant phytotoxicity of a group of MtNPs such as AgNPs, AuNPs, and CuONPs to certain plant species by inhibiting germination and root growth [173, 179, 180]. Different MtNPs have been assessed for plant toxicity based on their uptake, deposition and accumulation in plant cells or organs [25]. The results showed that the uptake and deposition of MtNPs depended on various factors including MtNP characteristics such as size, composition, surface characteristics, dose, delivery methods and plant species. The results also showed that bioaccumulation may affect plant physiology and plant growth [25, 181]. Deposition of MtNPs in the edible part of plants can cause a risk to human and animal health [173, 182, 183].

At the cellular level, MtNPs can enters to various organelles and interfere with the mitochondrial and chloroplast electron transport chains. In these cases they can activate metabolic pathways related to oxidative stress, which is associated with increased concentrations of reactive oxygen species and leads to cytotoxicity and genotoxic effects such as membrane damage, chlorophyll degradation, vacuole shrinkage, DNA damage and chromosomal aberrations [182, 184]. Excessive exposure of MtNPs to crop plants such as tomatoes, wheat, onions, etc. may cause oxidative bursts by interference with the electron transfer chain and can disrupt the ROS detoxifying, resulting in genotoxic implication. As a result, the production of secondary metabolites and phytohormones are affected and plant growth retardation occurs [25]. The phytotoxity and side effects of MtNPs that have been reported so far in crops include disturbances in water transfer, decreased photosynthetic rate, decreased growth hormone production, metabolic disorders, increased oxidative stress, chromosomal abnormalities, decreased growth, transcriptional changes in several genes and hypersensitivity to natural toxins such as arsenic [172, 185]. MtNPs can also affect beneficial plant-associated microbes in the surrounding soil when used to control phytopathogens. Microbes are associated epiphytically and endophytically with plants in the rhizosphere and soils near the plant root and may significantly promote plant growth through nitrogen fixation and phosphate solubilization [25, 186]. MtNPs used for plants crops and soil may have toxic effects on these beneficial microbes in the same way that they have on plant pathogens. These effects on the soil microbial community can be evaluated by measuring respiration and enzymatic activities in the soil [25]. For example, AgNPs have been shown to have potential antibacterial activity against soil microbial growth at levels below the concentrations of other heavy metals. Studies have also shown that AgNPs have toxic effects on beneficial microbial communities, including nitrogen-fixing bacteria, ammonifying bacteria and chemolithotrophic bacteria. These bacteria are able to form symbiotic relationship with leguminous plants and in addition to fixing nitrogen, affect plant yield and growth by secreting substances [175].

One of the main sources of indirect input of MtNPs, particularly AgNPs, is through discharge into wastewater which then leads to accumulation of these molecules into sewage sludge [173]. The main concern is the land application of this sewage sludge for agricultural or remediation purposes since the soil may receive a large source of silver contamination which can then affect plants and crops. Exposure of soil to MtNPs may lead to changes in microbial biomass, which in turn can affect plant growth and have physiological, biochemical, and molecular effects on them [172, 187]. With this risk of increased concentrations of potentially damaging materials, sustainable use of green synthesized NPs in agriculture will require further work to identify and address these issues. The development of less phytotoxic MtNPs must be examined in future studies and the effects of different MtNPs on plant growth at working concentrations must be determined coupled with clarification of the different effects of MtNPs application on plants and soil microbiota. Further research is also needed on the removal and clearance of MtNPs from agricultural soils and sewage sludge linked with experimental studies to understand the long-term effects of MtNPs on ecosystems and plant physiology.

## Conclusions

Green synthesis technology offers a potentially easy, efficient, clean, non-toxic and environmentally friendly method for the synthesis of MtNPs and has received much attention in recent years due to its economic prospects. A variety of microorganisms and plant extracts can be used for the efficient biosynthesis of MtNPs. While the synthesis of MtNPs using plants extracts is easier than that of microorganisms, the use of microorganisms to produce MtNPs is more cost-effective. Changing attitude of the international community towards sustainable development, improving environmental conditions and minimizing harmful man-made waste, provides a promising future for green synthesis of MtNPs and their application in various technologies, including agriculture.

Nanotechnology is an effective tool for improving the agricultural industry. The implementation of nanotechnology in modern agriculture, helps to boost the global economy. Given the various challenges posed by population growth and global climate change, the use of MtNPs in agriculture significantly helps to overcome the damage caused by excessive use of pesticides and chemical fertilizers for increasing crop production. More appropriate use of pesticides and fertilizers enclosed in various nanoformulations provides better application and controlled release and prevents environmental pollution. There are numerous studies on the successful use of various MtNPs in agriculture sector as nanobiosensors, nanopesticides and nanofertilizers. However, there is still not much knowledge about the adsorption capacity, permissible limit and environmental toxicity of these MtNPs.

Regardless of their origin as products with a specific purpose for agriculture as or the possibility of introducing them into the environment through the mismanagement of wastes containing MtNPs, it is necessary to carefully evaluate the toxicological effects of the MtNPs on the ecosystem. Therefore, in-depth studies are needed to investigate and determine their long-term effects, and if proven safe, they can be valuable as alternatives to conventional products used in agriculture. Nanotechnology is considered as one of the main components of sustainable agricultural development, but the promise of significant use of nanotechnology can only be achieved if ecotoxicity of these nanomaterials are fully assessed and properly managed.

## Data Availability

Not applicable.

## Abbreviations

MtNPs: Metal nanoparticles
AuNPs: Gold nanoparticles
AgNPs: Silver nanoparticle
MtONPs: Metal oxide nanoparticles
CF: Cell-free
CFE: Cell-free extract
FTIR: Fourier transform infrared
TEM: Transmission electron microscopy
SEM: Scanning electron microscopy
AFM: Atomic force microscopy
XRD: X-ray diffraction
EDS: Energy-dispersive spectroscopy
DLS: Dynamic light scattering
CdNPs: Cadmium NPs
CuNPs: Copper NPs
SeNPs: Selenium NPs
MG: Malachite green
CdSNPs: Cadmium sulfide NPs
LFIA: Lateral flow immunoassay
PLRV: Potato leaf roll virus
LSPR: Local surface plasmon resonance
CuONPs: Copper oxide NPs
PHSN: Hollow silica nanoparticles
SCP: Single cell protein
PdNPs: Palladium nanoparticles
GRAS: Generally recognized as safe
RP-HPLC: Reverse-phase-high performance liquid chromatography
GSP: Gold-shaped protein
ROS: Reactive oxygen species
